# Correlations between appraisals, management strategies, and psychological stress among schoolchildren with ADHD—A pilot study

**DOI:** 10.1002/jcv2.70106

**Published:** 2026-02-21

**Authors:** Noam Ringer, Adva Eichengreen

**Affiliations:** ^1^ Department of Special Education Stockholm University Stockholm Sweden; ^2^ Department of Special Education University of Haifa Haifa Israel; ^3^ Department of Developmental and Educational Psychology Leiden University Leiden Netherlands

**Keywords:** appraisals, attention‐deficit/hyperactivity disorder (ADHD), attributions, coping, self‐management, stress

## Abstract

**Background:**

Children with Attention‐Deficit/Hyperactivity Disorder (ADHD) encounter a range of difficulties in daily life. Guided by previous studies and theories, this pilot study examined whether distinct subgroups of children with ADHD could be identified based on their appraisals of the causes of their symptoms and the strategies they use to manage them. A further aim was to investigate associations between appraisals, management strategies, and psychological stress. Based on theory, we expected that appraisals attributing symptoms to external or controllable factors would be associated with lower stress levels.

**Methods:**

Ninety‐six children with ADHD, aged 9–16 years, completed questionnaires. The Children's Appraisals and Management of ADHD (CAM‐ADHD) questionnaire—developed for this study—measured appraisals of symptom causes and management strategies. Psychological stress was assessed using the Stress in Children questionnaire. Analyses focused on identifying underlying factors and examining correlations.

**Results:**

Three types of appraisals were identified: “ADHD is caused by biology/brain,” “ADHD is caused by the environment,” and “ADHD is part of personality.” Two types of management strategies emerged: “Attempting to exert self‐control” and “Asking for teachers' help.” Children who attributed their symptoms to environmental causes reported lower stress. Medication use was associated with greater use of self‐control strategies and reduced reliance on teachers' help.

**Conclusion:**

Children with ADHD differ in how they understand the causes of their symptoms and in the strategies they use to manage them. These differences are meaningfully related to psychological stress and medication use, underscoring the importance of considering children's own appraisals in assessment and intervention.

## Key Points

What's known?

- Schoolchildren with ADHD experience substantial daily challenges and psychological stress.
- Appraisals of symptoms and coping strategies are theoretically linked to stress but are rarely studied from schoolchildren's own perspectives.

**What's new?**

- Distinct patterns of symptom appraisals and management strategies were identified among schoolchildren with ADHD.
- Attributing symptoms to environmental causes was associated with lower stress, and medication use influenced coping strategies.

What's relevant?

- Schoolchildren's own understandings of ADHD are meaningfully related to stress and coping.
- Integrating schoolchildren's appraisals into assessment and intervention may enhance individualized support and well‐being.

## INTRODUCTION

Attention‐deficit/hyperactivity disorder (ADHD) is the most common mental health condition in childhood (Ayano et al., 2023) and is characterized by inattention, hyperactivity, and impulsivity (American Psychiatric Association, 2022). According to the DSM‐5‐TR, inattention involves difficulties sustaining focus and organizing tasks, reflected in careless mistakes, problems maintaining attention during academic activities, disorganization, and forgetfulness in daily routines (American Psychiatric Association, 2022). Hyperactivity refers to excessive motor activity or reduced ability to remain still, such as frequent fidgeting, leaving one's seat, running or climbing in inappropriate situations, and excessive talking. Impulsivity involves hasty actions without forethought, including interrupting others, difficulty waiting one's turn, or engaging in risky behaviors. These symptoms constitute a neurodevelopmental disorder when they are persistent, occur across multiple settings, and significantly interfere with social, academic, or other functional domains (American Psychiatric Association, 2022).

The manifestation of ADHD in children is highly heterogeneous (Biederman, 2004). ADHD is commonly divided into three subtypes: predominantly inattentive; predominantly hyperactive/impulsive; and combined presentation (American Psychiatric Association, 2022). Severity is further categorized as mild, moderate, or severe (American Psychiatric Association, 2022). Symptoms are also reported differently among boys and girls—boys more often display hyperactivity and impulsivity, while girls more frequently report inattention (Gershon, 2002). Symptom presentation also varies with age: younger children tend to show more hyperactivity and impulse‐control difficulties, whereas older children report more inattention (Lapalme et al., 2018; Willoughby, 2003).

Several researchers have suggested that there are individual variations also in how schoolchildren appraise the causes of their symptoms and the strategies they use to manage them, and that these variations have significant consequences (Koutsoklenis & Gaitanidis, 2017; Wong et al., 2019). For example, in a qualitative interview study, Ringer (2019) identified three ways in which children understand the causes of their ADHD: biological (brain‐based), environmental (related to external demands), and personality‐based (temperament). The study also identified variations in management strategies—some children attempted to control themselves, others adapted their environment, while a third group avoided demands or ignored symptoms. A similar variation was found by Wong et al. (2018), who reviewed both quantitative and qualitative studies and identified biological, environmental, and identity/personality‐based explanations among children with ADHD.

Some scholars aimed to understand the consequences of different cause‐explanations of mental illness for the individual's well‐being and treatment outcomes. Not specifically for schoolchildren with ADHD, Lebowitz (2014) has conducted a review of the consequences of endorsing biomedical explanations for mental disorders among affected individuals. The review has found that individuals who perceive the causes of their mental illness in terms of biomedical explanations tend to have lower perceived ability to manage their illness, lower motivation to manage challenges, and less hope with regards to treatment's prognoses (Lebowitz, 2014). However, the review has illuminated that there is evidence of also beneficial consequences for the biomedical explanation, as it tends to reduce self‐blame and stigma (Lebowitz, 2014).

Specifically for schoolchildren with ADHD, Wong et al. (2019a) investigated the correlations between various explanations of ADHD, strategies for managing symptoms, perceived quality of life, and treatment adherence. The study has found that beliefs that ADHD is caused by biomedical mechanisms and beliefs that psychological and environmental factors are not the causes of ADHD were related to attempts to control or minimize the symptoms as well as to a higher perceived quality of life. With regard to adherence to medication, no relationships were found between adherence and beliefs (Wong et al., 2019b). However, in another study, it was found that beliefs that ADHD is the result of a biological dysfunction were significant predictors of medication adherence (Emilsson et al., 2017). Regarding the possible implications of appraisals and management strategies, Wong et al. (2019a) examined the relationships between perceptions of ADHD, management strategies, quality of life, and treatment adherence among youths aged 10–18. The results showed that beliefs in biological causes and disbelief in environmental or psychological factors were associated with attempts to control or reduce symptoms and higher perceived quality of life. Conversely, beliefs that ADHD is highly impactful and difficult to control were related to avoidant coping and lower quality of life. No link was found between beliefs and medication adherence in this study. However, Emilsson et al. (2017) showed that beliefs in biological causes did predict medication adherence in a separate sample.

### Theoretical framework

This study was based on two theoretical frameworks: the Transactional Theory of Stress and Coping (Lazarus & Folkman, 1984) and the Mixed‐Blessings Model (Haslam & Kvaale, 2015). Together, they support assumptions about the links between appraisal, coping, and stress and inform predictions about relationships between biomedical explanations of ADHD and perceived stress.

According to the Transactional Theory of Stress and Coping, individuals interpret events to determine whether they pose a threat to well‐being, based on both characteristics of the event and personal factors such as goals, values, and beliefs (Lazarus & Folkman, 1984). Threat interpretations commonly trigger emotional distress. The Transactional Theory further proposes three categories of coping strategies. Emotion‐focused coping aims to regulate emotional responses (e.g., self‐control). Problem‐focused coping targets the stressor directly, such as seeking support or adjustments. Disengaged coping involves distancing oneself from the stressor, such as ignoring or withdrawing from demands (Biggs et al., 2017).

The Mixed‐Blessings Model (Haslam & Kvaale, 2015) proposes that biomedical explanations may have dual emotional consequences. On one hand, biological explanations reduce self‐blame and social stigma by locating symptoms outside the individual's control. On the other hand, they may also reduce perceived agency, increase beliefs that symptoms are uncontrollable, and heighten withdrawal or resignation. This model also connects biomedical explanations to psychological essentialism, where symptoms are seen as a fixed, defining quality of the self. Essentialism may increase pessimism about change, yet may also promote self‐acceptance by framing symptoms as part of one's identity rather than a personal failing.

### Summary and rationale

Together, the empirical findings and theoretical models highlight the complex relationships between individuals' interpretations of their ADHD symptoms, their coping strategies, and their emotional well‐being. Biomedical explanations of mental disorders appear to be neither uniformly beneficial nor uniformly harmful: they may reduce self‐blame, but can also diminish perceived controllability and self‐efficacy. The Transactional Theory of Stress and Coping (Lazarus & Folkman, 1984) suggests that such appraisals shape coping responses, while the Mixed‐Blessings Model (Haslam & Kvaale, 2015) emphasizes that biomedical explanations may simultaneously reduce self‐blame and reduce perceived control. Empirical studies reflect these mixed outcomes: biological explanations can relate to self‐control strategies and, in some cases, higher quality of life, but may also undermine perceived agency or relate differently to treatment adherence (Emilsson et al., 2017; Lebowitz, 2014). These variations in findings motivate the present study's focus on identifying appraisal and coping profiles among school‐aged youth with ADHD and examining how these patterns are associated with perceived stress, with the aim of contributing additional knowledge to this area.

## RESEARCH QUESTIONS, AND HYPOTHESES

This study aimed to address the following research questions:

1. Are there distinct groups of schoolchildren with ADHD based on their appraisals of the causes of their symptoms and the strategies they use to manage them? Guided by prior research and theory, we anticipated three appraisal profiles—biomedical, environmental, and personality‐based—and three management profiles reflecting schoolchildren’ tendencies to control themselves, adapt their environment, or avoid demands.
2. What are the associations between causal appraisals, management strategies, and self‐perceived psychosocial stress? We expected significant relationships among these variables; however, we did not anticipate specific directional hypotheses due to the mixed findings in previous literature.
3. Are appraisals and management strategies associated with participants' characteristics (age, gender, additional diagnoses, and medication use), and what is the nature of these associations? We hypothesized non‐directional significant correlations.

## METHOD

### Study design

This study applied a cross‐sectional correlational design, which is suitable for examining relationships among variables at a single point in time.

### Participants

Participants were schoolchildren diagnosed with ADHD. Of the 97 participants who completed the questionnaires, one was excluded due to incomplete responses, resulting in a final sample of 96 participants (43 boys), aged 9–16 years (*M* = 13.13, SD = 1.73). Table 1 presents the sample characteristics.

**TABLE 1:** Demographic characteristics of participants.

No. of participants	96
Gender—*n* (%)	
Identifies as a girl	51 (53.1)
Identifies as a boy	43 (44.8)
Other gender identity	2 (2.1)
Additional diagnosis—*n* (%)	
No	64 (66.7)
Autism	6 (6.3)
Language disorder or dyslexia	23 (24)
Don't want to answer	3 (3.1)
Medication—*n* (%)	
No	20 (20.8)
Sometimes	15 (15.6)
Every day	61 (63.5)
Mean age in years (SD); range	13.13 (1.73); 9–16
Mean age in years at ADHD diagnosis (SD); range	10.99 (2.14); 4–15

### Instruments

Data were collected using the Children's Appraisals and Management of ADHD (CAM‐ADHD)—a questionnaire developed for this study—along with the Stress in Children (SiC) questionnaire and demographic and medical background questions.

#### Children’s appraisals and management of ADHD

The CAM‐ADHD was developed to measure how children with ADHD appraise the causes of their symptoms and the strategies they use to manage them. Development followed the procedure outlined by Davis (1992). First, an item pool of 80 statements was generated reflecting different causal appraisals and management strategies. Based on previous research (Ringer, 2019; Wong et al., 2018), items covered three appraisal domains (biological, environmental, and personality‐based causes) and three forms of symptom management (self‐control, seeking teacher adjustment, and withdrawal).

An independent panel of five field experts (two psychologists, two special educators, and one occupational therapist), recruited through professional networks, rated the relevance of each item on a four‐point scale (1 = not relevant, 2 = somewhat relevant, 3 = quite relevant, 4 = very relevant) (Davis, 1992). Twenty‐four statements rated as very relevant by all raters were selected for the preliminary version of the instrument.

To assess comprehensibility, five children aged 10–16 years with ADHD were interviewed. They were asked to explain each item in their own words. Items that were unclear were reformulated.

All items refer to the school context. Examples of biological appraisals include “I lose my patience because of my brain” and “I'm restless because of my brain.” Environmental appraisal items include “I'm restless because the classroom is messy” and “I lose my patience because other people talk for too long.” Personality‐based appraisal items include “I'm restless because that's who I am as a person” and “I lose my patience because that's who I am as a person.”

Self‐control management items include “If I'm restless in class, I try to control myself even more” and “If I lose focus when working in class, I make an effort to stay focused.” Items representing help‐seeking include “If I'm restless in class, I ask my teacher for help” and “If I lose focus when working in class, I ask my teacher for help.” Withdrawal‐based management is reflected in items such as “If I'm restless in class, I walk around or leave the classroom” and “If I lose focus when working in class, I ignore the assignment.” All subscales' total scores are computed as mean scores. The instruction for appraisal items was: “Here are some statements that describe different ways of thinking. How often do YOU think in these ways? Choose from: Never, Sometimes, Often, Always.” The instruction for management items was: “Here are some statements that describe different ways of reacting. How often do YOU react in these ways? Choose from: Never, Sometimes, Often, Always.” The full questionnaire is available in the Supporting Information (Appendix S1).

#### Stress in children

The SiC questionnaire (Osika et al., 2007) consists of 21 self‐report items measuring stress‐related experiences. Items use four Likert‐type response options: Never, Sometimes, Often, and Always. The instrument includes physical symptoms (e.g., “I have a headache,” “I have a stomachache”), emotional experiences (e.g., “I get angry,” “I feel happy”), and social experiences (e.g., “When things are hard, there's an adult I can talk to,” “When things are hard, it helps to be with my friends”). The SiC was developed and validated in Sweden and demonstrates good psychometric properties (Osika et al., 2007). In this study, internal reliability was acceptable (Cronbach Alpha = 0.791).

### Procedure

All procedures in the study were performed in accordance with ethical standards, and have been reviewed and approved by the Swedish Ethics Review Authority (Registration number 2021‐01004).

To achieve variation in the sample, recruitment was conducted through multiple channels, including special educators in schools as well as nurses and clinical psychologists working in child and adolescent medical clinics across Stockholm. Participants were eligible for inclusion if they had a formal ADHD diagnosis and were able to read and understand Swedish. Potential participants and their parents received written information about the study when their appointment was scheduled, and written informed consent was obtained from parents and from children aged 15 years or older. Most participants were recruited through child and youth medical clinics (71 participants; 73% of the sample). Children completed the questionnaires on site, and families were assured anonymity and informed of the child's right to withdraw from the study or omit any item. Completed questionnaires and consent forms were then collected by the first author.

### Data analysis

Data were analyzed using SPSS (Version 27; IBM Corp.). The analytical process comprised several consecutive steps. First, descriptive analyses were conducted to identify outliers, examine missing data, and inspect the distributional characteristics of all variables. Internal consistency for each scale was assessed using Cronbach's alpha. To identify potential clusters in causal appraisals and management strategies, principal components analysis was performed, as this method allows for the detection of underlying component structures. Item‐level analysis was also conducted, including interitem correlations and examinations of variance and response frequencies, to identify redundant or invariant items.

Associations between causal appraisals, management strategies, and stress were examined using Spearman's rho correlations between the CAM‐ADHD subscales and the SiC total scores, with statistical significance set at *p* (two‐tailed) < 0.05. Finally, to explore factors potentially associated with appraisals and management strategies, five linear regression analyses were conducted with the CAM‐ADHD subscale scores as dependent variables and age, gender, additional diagnoses, and medication use entered as predictors.

## RESULTS

The results are presented in accordance with the three research questions. First, we describe the identification of distinct profiles among schoolchildren with ADHD based on their appraisals of symptom causes and the strategies they employ to manage them. Next, we report the associations between these appraisal and management profiles and self‐perceived psychosocial stress. Finally, we examine how appraisals and management strategies relate to participant characteristics, including age, gender, additional diagnoses, and medication use.

### Identification of distinct profiles among schoolchildren with ADHD

**FIGURE 1:**
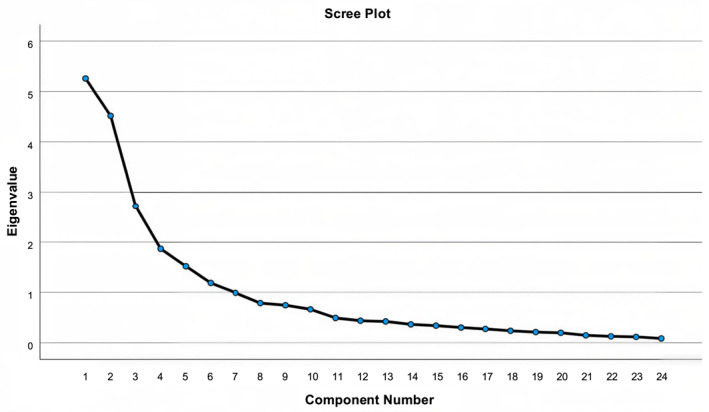
Scree plot of the factors' eigenvalues regarding the CAM‐ADHD questionnaire.

To examine clustering of participants according to their appraisals and management of ADHD manifestations, a Principal Component Factor Analysis was conducted. Bartlett's test of Sphericity was statistically significant (*χ* ^2^ = 1345.003, *p* < 0.001), showing satisfactory correlation among variables. Sample adequacy was confirmed by a Kaiser‐Meyer‐Olkin test value of 0.728 (>0.5). The analysis indicated six factors with eigenvalues of ≥1, which combined accounted for 71.09% of the variance. Figure 1 describes the scree plot and indicates that these six factors should be retained.

The following factors were identified: Factor 1, “Causality: Biological,” includes items that refer to appraisals that the ADHD symptoms are caused by the participant's brain; Factor 2, “Causality: Environment,” includes items that refer to appraisals that the ADHD symptoms are caused by inappropriate conditions in the environment; Factor 3, “Causality: Personality,” includes items that refer to appraisals that the ADHD symptoms are caused by one's personality; Factor 4, “Management: Efforts at self‐control,” includes items that describe management of ADHD by self‐regulation, either by making efforts to control oneself or (oppositely scored) by releasing control. In this factor, all four items depicting self‐control correlated negatively with the four items depicting releasing control; Factor 5, “Management: Asking teacher for adjustments,” includes items that refer to management of difficulties by asking teachers to adjust demands or assignments; and Factor 6, “Forget‐Ask,” includes only one item, which refers to management of forgetfulness by asking teachers or friends about the forgotten information. After the analysis we removed this final item; thus, the final questionnaire that was used in the rest of the analysis consisted of 23 items grouped into five subscales. The final results of the factor analysis and the subscales' internal consistencies are displayed in Table 2. Subscales' internal consistencies range from acceptable to good.

**TABLE 2:** Subscales' internal consistencies.

Item	Causality: Biological	Causality: Environment	Causality: Personality	Management: Self‐control	Management: Ask for help
Lose focus because of my brain	0.905				
Restless because of my brain	0.861				
Lose patient because of my brain	0.755				
Forget because of my brain	0.709				
If lose focus I ignore				0.866	
If restless I walk around or leave				0.828	
If restless homework I ignore				0.805	
Restless homework control				−0.757	
If lose focus in class I make effort				−0.676	
If forget I try hard remember				−0.663	
If forget I ignore				0.642	
If restless in class I control myself				−0.560	
Restless because class is messy		0.766			
Lose focus because teachers boring		0.733			
Impatient because other talk too long		0.690			
Forget because too many tasks		0.688			
If restless ask teacher for help					−0.825
If lose focus ask teacher for help					−0.751
If restless with homework I ask teacher for different/fewer					−0.746
Lose focus because of who I am as a person			−0.909		
Restless because of who I am as a person			−0.855		
Forget because of who I am as a person			−0. 791		
Impatient because of who I am as a person			−0.675		
Chronbach alpha (no. of items)	0.888 (4)	0.749 (4)	0.861 (4)	0.888 (8)	0.716 (3)

*Note*: Rotation method: Oblimin with Kaiser Normalization. Rotation converged in 10 iterations.

#### Correlations between subscales

Component correlation matrix is presented in Table 3, and Spearman bivariate correlations between the subscales' Mean scores are displayed in Table 4. One significant correlation was found between biological appraisal of causality and personality appraisal of causality (rs = 0.49, *p* < 0.001).

**TABLE 3:** Component correlation matrix for the CAM‐ADHD five factors.

	Causality: Biological	Management: Self‐regulation	Causality: Environment	Management: Ask for help	Causality: Personality
Causality: Biological	_	_	_	_	_
Management: Self‐regulation	−0.052	_	_	_	_
Causality: Environment	0.107	−0.070	_	_	_
Management: Ask for help	−0.110	−0.116	−0.116	_	_
Causality: Personality	−0.366	0.029	−0.111	0.038	_

**TABLE 4:** Spearman correlations between the CAM‐ADHD five factors' mean scores.

	Causality: Biological	Causality: Environment	Causality: Personality	Management: Self‐control	Management: Ask for help
Causality: Biological	_	_	_	_	_
Causality: Environment	0.052	_	_	_	_
Causality: Personality	0.492***	0.121	_	_	_
Management: Self‐control	−0.35	−0.117	−0.083	_	_
Management: Ask for help	0.16	0.175	−0.011	0.138	_

***p < 0.001.

#### Item analysis

To further examine the psychometric properties of the CAM‐ADHD scale, a correlation matrix of all CAM items was conducted and is available in the Supporting Information (Appendix S2). Item intercorrelations were acceptable and did not indicate item redundancy. Items were examined for their variance and frequency of responses. All items showed the full range of responses (1–4). Additionally, all items showed strong variance (STD ≈ 1 for most items, and ≈0.8 for the three “Asking for Help” items). Distribution of responses for each item did not exceed 40% for most items, except for the “Asking for Help” items, which was still acceptable (highest frequencies range 45%–57% for “Never”).

### Associations between appraisals and management strategies

To examine whether certain appraisals were associated with certain management strategies, Spearman's Rho correlations were conducted between the CAM‐ADHD subscales. No significant correlations were found.

### Associations between appraisals, management strategies and stress

To examine whether certain appraisals and strategies were related to stress, Spearman Rho correlations were conducted between the CAM‐ADHD subscales and the SiC. A significant correlation was found between the “Causality: Environment” subscale and SiC (Spearman's Rho = 0.211, *p* = 0.040), meaning that attributing the causes of symptoms to environmental conditions was associated with less stress.

Stratifying the correlations by gender revealed a significant correlation for boys between self‐control management and SiC (Rho = 0.451, *p* = 0.002), suggesting that, only for boys, ignoring or leaving difficult tasks instead of trying to self‐control was related to less stress.

### Participants' characteristic related to appraisals and management

To examine whether appraisals and managements were related to participants' characteristics including age, gender, additional diagnosis, and medications, we conducted five linear regressions with the five CAM‐ADHD subscales as dependent variables and participant characteristics as the predictors. The responses for the variables Gender and Additional diagnosis were recoded to include two categories. In the Additional diagnosis variable, the response “Don't want to answer” was recoded to missing value.

Table 5 presents the regression weights of the models that were found to be significant. Causality subscales (Biological, Environment, and Personality) and the “Asking for Help” management did not present significant models and are thus not reported.

**TABLE 5:** Regression weights (standard error) for variables predicting CAM‐ADHD subscales.

Parameter	Management self‐regulation		Management asking for help	
	Unstandardized B (SE)	*p* value	Unstandardized B (SE)	*p* value
Intercept	1.865 (0.769)	0.017	1.795 (0.672)	0.009*
Age	0.063 (0.049)	0.205	0.006 (0.043)	0.888
Gender	−0.051 (0.160)	0.752	0.115 (0.140)	0.415
Medication	−0.256 (0.097)	0.010*	−0.236 (0.086)	0.006*

For the subscale “Management: Efforts at control”, the overall regression model was statistically significant (*R* ^2^ = 0.138, *F* (4, 88) = 3.522, *p* = 0.010), with medication significantly predicting fewer leave (ignore) and more self‐control strategies.

## DISCUSSION

This study examined the relations between schoolchildren's’ appraisals of the causality of their ADHD symptoms, their management strategies, and their psychological stress. Firstly, we found distinct patterns of causality appraisal (biological, environmental, personality) and management strategies (attempting for self‐control, asking for teacher's help) that schoolchildren with ADHD may apply. These findings confirm results of previous qualitative research (Ringer, 2019; Wong et al., 2018). Interestingly, attributing causality to biology was moderately associated with attributing causality to one's personality, suggesting that biology is often perceived as an essential part of one's identity. Yet, this association was not full. An example for unrelatedness between these appraisal categories could be a person who perceives their brain function as external to who they “really are.” Future qualitative research could deepen our understanding of the various ways ADHD is perceived in relation to adolescent's sense of identity, or in relation to how they wish to define themselves. It should be noted that the correlations between appraisals and management styles were insignificant, suggesting that bivariate correlations may not be sensitive enough to fully capture the distinction between aspects of appraisal and management; or alternatively that they may not highly correlate, for example, when children can hold several appraisals and management strategies that are applied in different contexts.

Identifying variation among children with ADHD in terms of their appraisals and management strategies may have important clinical implications. In a case in which a child appraises the cause of their ADHD to be environmental conditions or their personality, while the clinicians and parents consider pharmacological treatment, not considering the child's appraisal may result in low treatment adherence and relational conflicts. The CAM‐ADHD can be used as a reliable instrument for identifying patients' views on causes, as a point of departure for clinical work.

A further finding of the study was that children who viewed the causes of their ADHD symptoms to be conditions in the environment experienced less stress. This finding has a different direction from the association found by Wong et al. (2019b), who found that beliefs that ADHD is caused by biological factors rather than environmental ones were related to a higher perceived quality of life. An explanation for the dissimilarities in the results could be that our study measured psychological stress while Wong et al. (2019a) measured perceived quality of life. However, understanding this result through the lens of the Transactional Theory (Lazarus & Folkman, 1984), we could hypothesize that appraising ADHD as a result of environmental factors may be experienced as less threatening than viewing the symptoms as experiences from within oneself. It could also be assumed that environmental causes may be perceived as more manageable, or that this perspective preserves a positive self‐perception. From the perspective of the social model of disability (Oliver, 1990), disability is caused by environmental and interpersonal barriers that disable persons with impairments and hamper their participation in society, including their emotional well‐being (Reeve, 2004; Thomas, 1999). For children with disabilities in particular, understanding the source of difficulties as stemming from the environment (e.g., teachers' and peers' attitudes, educational and classroom adaptations) rather than the child significantly influences the child's self‐perception and may alleviate feelings such as self‐blame, internalized ablism, and psychological stress (Eichengreen & Hoofien, 2020; Hunt, 2024).

Professionals and clinicians often emphasize the child's resources to adapt and cope with environmental demands, including self‐advocacy skills. However, Stuntzner and Hartley (2015) highlighted the emotional toll which may accompany such coping endeavors, such as exhaustion and coping with other people's reactions, which may lead to internalized stigma and self‐criticism. Educators, clinicians and parents need therefore to acknowledge the inherent difficulty in trying to adapt to neurotypical environments and assist schoolchildren with ADHD in developing self‐compassion skills (Stuntzner & Hartley, 2015), which may alleviate daily stress. A community‐based approach, which connects between neurodivergent individuals, for example, via online platforms or support groups, may be particularly beneficial for schoolchildren who study in mainstream classes to develop self‐affirmative approach to ADHD‐related needs (Hunt, 2024). This affirmative approach aligns with current perspectives on neurodiversity as a natural and valuable aspect of human variation, brought about by the neurodiversity and critical ADHD studies movements (Dwyer, 2022; Jackson‐Perry et al., 2025). Additionally, reframing coping from an individual perspective to interpersonal one (e.g., dyadic coping; Bodenmann et al., 2018; Eichengreen et al., 2025) can assist in developing supportive networks were peers and teachers adapt to neurodivergent needs as part of the ordinary classroom schedule, instead of referring it as an individual “problem” of the child with ADHD. These steps would alleviate stress and increase students' sense of belonging.

This result has also clinical implications with regards to educational interventions to promote the notion that biological factors involved in the etiology of ADHD. Such interventions are malleable and responsive to individual actions. Emphasizing treatability and malleability may reduce negative prognostic beliefs, whereas biomedical explanations often promote essentialist views of mental disorders as biologically determined and immutable. Integrating a balanced biopsychosocial–societal perspective into educational interventions would provide a fuller account of its complex and heterogeneous etiology and support the development of more diverse and contextual understanding of the schoolchildren’ lives with ADHD.

An additional finding of the study is that boys who tend to abandon difficult tasks rather than persist in exerting self‐control experience lower levels of stress. This finding may be related to gender differences in the manifestation of ADHD, with boys typically exhibiting higher levels of hyperactivity and impulsivity (Gershon, 2002). These characteristics may contribute to lower environmental expectations and, consequently, lower self‐expectations regarding sustained effort on demanding tasks. Stress levels may also be reduced among boys who relinquish control due to differential gender expectations in education, where girls are often expected to be more compliant (Jones & Myhill, 2004).

Another potential explanation can be presented from a social model perspective: If one has hyperactivity and impulsivity, which is often the case for boys, a way to deal with this is not to try to control it but to let it go (e.g., go for a walk to release energy) instead of trying to conform to neurotypical standards. Similarly, previous research has indicated that applying problem solving strategies which are directed at changing the stressor may be ineffective for children's well‐being when stressors were perceived as uncontrollable. This was found for schoolchildren belonging to minority groups (Vera et al., 2011), those having chronic illness (Compas et al., 2012) or autism (Pouw et al., 2013), those experiencing peer victimization (Cooley et al., 2022), or deaf or hard‐of‐hearing children whose classmates do not adapt to their hearing‐related needs (Eichengreen et al., 2025). In such cases, accepting the stressor and trying to create a cognitive distance from it (e.g., Compas et al., 2012; Cooley et al., 2022) may be more adaptive for the child's well‐being in terms of stress level. This may also be the case for schoolchildren with ADHD who try to take “time off.” However, these hypotheses should be further investigated in future studies.

Furthermore, the results showed that children who take medication tend to control themselves more. Possibly, the effect of ADHD medication increases a child's ability to for self‐control and consequently encourages more utilization of this coping strategy.

### Methodological limitations and suggestions for future research

The present study has several methodological limitations that warrant consideration. First, the relatively small sample size constrains the validity of the findings and limits the conclusions that can be drawn regarding the psychometric properties of the newly developed instrument. Due to ethical considerations regarding secure data protection the participants have filled in the questioners on place in paper form. As it was pointed by other researchers (e.g., Rice et al., 2019), this way of recruitment technique, although beneficial from an ethical perspective, is challenging. A primary challenge was limited engagement among the schoolchildren, who were often reluctant to invest the time and effort required for participation. Additionally, parental engagement posed a further challenge; many parents were unable or unwilling to encourage their child's participation. Because recruitment was conducted in connection with clinical appointments, parents accompanying their child often did not have the time to remain at the clinic for the duration needed to complete the study procedures. Finally, limited engagement and available time among clinic staff also impeded the recruitment process, as staff reported difficulties integrating recruitment activities into their routine workflow. These recruitment‐related limitations highlight the need for future studies to develop and evaluate more effective strategies for engaging schoolchildren with ADHD and their families in research. Further investigation is warranted to identify approaches that reduce participant burden, increase motivation, and facilitate collaboration with clinical staff, in order to ensure adequate sample sizes to further assess the psychometric properties of the new questionnaire.

Secondly, data was not collected on either the subtype or severity of the ADHD. The analysis is mainly interested in the children's own perceptions and management of their ADHD, but it is possible that medical aspects regarding the diagnosis itself play a role in the relations between appraisals, management, and stress.

A third limitation is that, despite efforts to reach variation in how participants were recruited to the study, the majority were recruited via child and youth clinics. This may have affected the generalizability of the results—that is, the group who has contact with medical healthcare may be overrepresented in the sample. Also, an additional psychiatric diagnosis of anxiety or mood disorder may have relevance for the experiences of children and youth with ADHD (Jackson et al., 2023). However, with regard to the use of ADHD medication and comorbidity with another neurodevelopmental diagnosis, the sample is representative of the population (Socialstyrelsen, 2024).

Furthermore, since the data consider experiences in the school context it would be reasonable to assume that school characteristics, such as the type of school and aspects of the learning environment in the classroom, may contribute to the understanding of using management strategies and stress (Gibbs et al., 2020; Kulawiak, 2021). As the same child may hold different appraisals and management strategies, as indicated in this study, future research would benefit from exploring the contexts that elicit intra‐ and inter‐personal differences in the application of appraisals or strategies.

## AUTHOR CONTRIBUTIONS

**Noam Ringer:** Conceptualization; funding acquisition; investigation; methodology; validation; project administration; writing—review and editing; writing—original draft; **Adva Eichengreen:** Investigation; writing—original draft; writing—review and editing; formal analysis.

## CONFLICT OF INTEREST STATEMENT

The authors declare no conflicts of interest.

## ETHICAL CONSIDERATIONS

Written informed consent was obtained from parents and from schoolchildren aged 15 years or older, in accordance with ethical requirements for research involving minors. Ethical approval for the study was granted by the Swedish Ethics Review Authority (Etikprövningsmyndigheten). The approval was issued on 31 December 2021, under registration number 2021‐01004.

## Supporting information

Supporting Information S1

Supporting Information S1

Supporting Information S2

## Data Availability

The data that support the findings of this study are openly available in Swedish National Data Service (SND) at https://doi.org/10.58141/gcfa‐tp98, reference number SU‐317‐0056‐23.
