# Supplementary material for: Correlations between appraisals, management strategies, and psychological stress among schoolchildren with ADHD—A pilot study
Source: JCPP Adv. 2026 Feb 21;6(3):e70106. doi: 10.1002/jcv2.70106 (PMC13339038; doi:10.1002/jcv2.70106)
Supplement: Supplementary file 1 [file JCV2-6-e70106-s001.docx]

**Correlations between appraisals, management strategies, and psychological stress among schoolchildren with ADHD- a pilot study**

**Supporting Information**

**Appendix S1**. The Children’s Appraisals and Management of ADHD (CAM-ADHD) questionnaire

| Here are some statements that describe different ways of **thinking**. How often do YOU **think** these ways? Choose from: **Never** \| **Sometimes** \| **Often** \| **Always** | | | | | |
| --- | --- | --- | --- | --- | --- |
|  | | **Never** | **Sometimes** | **Often** | **Always** |
| **1** | I lose focus **because** **the teachers are boring.** |  |  |  |  |
| **2** | I lose focus **because** of **my brain**. |  |  |  |  |
| **3** | I lose focus **because** **that’s who I am as a person.** |  |  |  |  |
| **4** | I’m restless **because the classroom is messy.** |  |  |  |  |
| **5** | I’m restless **because of my brain.** |  |  |  |  |
| **6** | I’m restless **because that’s who I am as a person.** |  |  |  |  |
| **7** | I forget what to do **because there are too many things to do**. |  |  |  |  |
| **8** | I forget what to do **because of my brain.** |  |  |  |  |
| **9** | I forget what to do **because that’s who I am as a person.** |  |  |  |  |
| **10** | I lose my patience **because other people talk for too long.** |  |  |  |  |
| **11** | I lose my patience **because of my brain.** |  |  |  |  |
| **12** | I lose my patience **because that’s who I am as a person.** |  |  |  |  |
| Thank you! You're almost halfway there! | | | | | |
| Here are some statements that describe different ways of **reacting**. How often do YOU **react** these ways? Choose from: **Never** \| **Sometimes** \| **Often** \| **Always** | | | | | |
|  |  | **Never** | **Sometimes** | **Often** | **Always** |
| **13** | If I’m restless in class, I **try to control myself even more.** |  |  |  |  |
| **14** | If I’m restless in class, **I ask my teacher** for help. |  |  |  |  |
| **15** | If I’m restless in class, **I walk around** or leave the classroom. |  |  |  |  |
| **16** | If I lose focus when working in class, I **make an effort** to stay focused. |  |  |  |  |
| **17** | If I lose focus when working in class, **I ask my teacher for help.** |  |  |  |  |
| **18** | If I lose focus when working in class, **I ignore the assignment.** |  |  |  |  |
| **19** | If I forget what I’m supposed to do, **I try hard to remember.** |  |  |  |  |
| **20** | If I forget what I’m supposed to do, **I ask my teacher or friends.** |  |  |  |  |
| **21** | If I forget what I’m supposed to do, **I ignore what I’m supposed to do.** |  |  |  |  |
| **22** | If I feel restless when doing homework, **I try hard to continue.** |  |  |  |  |
| **23** | If I feel restless when doing homework, **I ask my teacher for different/less homework.** |  |  |  |  |
| **24** | If I feel restless when doing homework, **I ignore the homework** and do something else instead. |  |  |  |  |
| **Thank you for your help!** | | | | | |
