# Supplementary material for: Correlations between appraisals, management strategies, and psychological stress among schoolchildren with ADHD—A pilot study
Source: JCPP Adv. 2026 Feb 21:e70106. Online ahead of print. doi: 10.1002/jcv2.70106 (PMC13339038; doi:10.1002/jcv2.70106)
Supplement: Supplementary file 1 — Supporting Information S1 [file JCV2-9999-e70106-s001.docx]

**Supplementary Materials**

**Appendix S1**. The Children’s Appraisals and Management of ADHD (CAM-ADHD) questionnaire

**Appendix S2**. A correlation matrix of all CAM items
